# Supplementary material for: Identification of Freezing-Responsive microRNAs and Their Targets in Chinese Jujube by Small RNA and Degradome Sequencing
Source: Int J Mol Sci. 2026 Sep 4;27(17):7912. doi: 10.3390/ijms27177912 (PMC13566890; doi:10.3390/ijms27177912)
Supplement: Supplementary file 1 [file ijms-27-07912-s001.zip › Supplementary Material S2/novel_miR_170.pdf]

Provisional ID	:	novel_miR_170
Score total	:	900.9
Score for star read(s)	:	3.9
Score for read counts	:	894.4
Score for mfe	:	1
Score for randfold	:	1.6
Score for cons. seed	:	
Total read count	:	1766
Mature read count	:	1748
Loop read count	:	0
Star read count	:	18

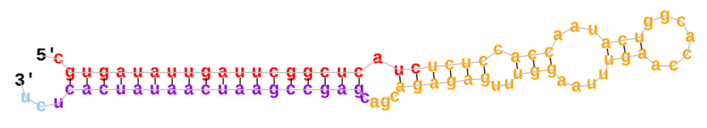

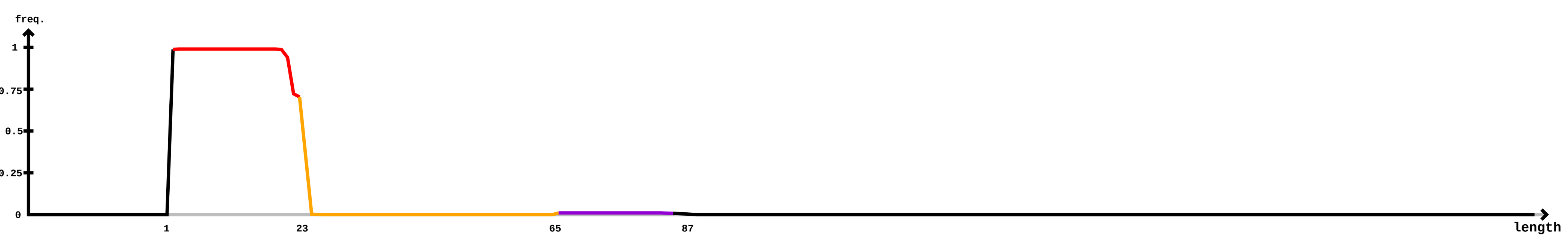

Mature Star

```
5'- cauucugcagaaguagacauggcgugauauugauucggcucaucucuccaccaauacuggcaccaaguuuaagguuugagagacgacgagccgaaucaauaucacucuuguaugcuucuucgcauauuaucaagguuuuuccuguuuuuugaguuccaauauuagccaagaagcaucucugcaggcaacucaugcauuaagcuacauagaagcuuguuuuggcucuccaucccccucuuguaaagucuuc -3' obs
    cauucugcagaaguagacauggcgugauauugauucggcucaucucuccaccaauacuggcaccaaguuuaagguuugagagacgacgagccgaaucaauaucacucuuguaugcuucuucgcauauuaucaagguuuuuccuguuuuuugaguuccaauauuagccaagaagcaucucugcaggcaacucaugcauuaagcuacauagaagcuuguuuuggcucuccaucccccucuuguaaagucuuc     exp
    ....((((((........((.((((((((((((((((.((((((.(((...(((......)))....)))..))))))....)))))))))))))))).)).((((((.....))))))......((.....))((((((((.(((.......)))))))))))..)))))))(((.....)))..((((((......))))))....((((...((.........))...))))...   reads  mm  sample
    ......................cgugauauugauucggcu          5    0   LT-3
    ......................cgugauauugauucggcu          1    0   LT-1
    ......................cgugCuauugauucggcuc         1    1   LCK-3
    ......................cgugauauugauucggcuc         7    0   LT-1
    ......................cgugauauugauucggcuc         13   0   LT-3
    ......................cgugauauugauucggcuU         1    1   LT-3
    ......................cgugauauugauucggcuc         21   0   LT-2
    ......................cgugauauugauucggcuc         33   0   LCK-3
    ......................cgugauauugauucggcuA         6    1   LCK-3
    ......................cAugauauugauucggcuca        1    1   LT-2
    ......................cgugauauugauucggcuca        60   0   LCK-2
    ......................cgugauauugauucggcuca        3    0   JCK-3
    ......................cgugauauugauucggcuca        3    0   JT-3
    ......................cgugauauugauucggcuca        23   0   LT-1
    ......................cgugauauugauucgAcuca        1    1   LCK-3
    ......................cgugauCuugauucggcuca        1    1   LT-3
    ......................cgugauauugauucggcuca        52   0   LT-3
    ......................cgugauCuugauucggcuca        1    1   LCK-2
    ......................cgugauauugauucggcuca        103  0   LCK-3
    ......................cgugauauugauucggcucU        3    1   LT-2
    ......................cgugauauugauucggcuca        9    0   JCK-2
    ......................cgugauauugauucggcuca        2    0   JCK-1
    ......................cgugauauugauucggcuca        1    0   JT-2
    ......................cgugauauugauucggcuca        71   0   LT-2
    ......................cgugauauugauucggcuca        48   0   LCK-1
    ......................cgugauCuugauucggcuca        1    1   LCK-1
    ......................cgugauauugauucggcucau       1    0   LT-3
    ......................cgugauauugauucggcucau       8    0   LT-1
    ......................cgugauauugauucggcucau       9    0   LT-2
    ......................cgugauauugauucggcucau       14   0   LCK-3
    ......................cgugauauugauucggcucaA       1    1   LT-3
    ......................cgugauCuugauucggcucau       1    1   LT-3
    ......................cgugauauugauucggcucauU      5    1   LT-3
```

```
                      Mature                                                          Star
cauucugcagaaguagacauggcgugauauugauucggcucaucucuccaccaauacuggcaccaaguuuaagguuugagagacgacgagccgaaucaauaucacucuuguaugcuucuucgcauuauucaagguuuuuccuguuuuuugaguuccaauauuagccaagaagcaucucugcaggcaacucaugcauuaagcuacauagaagcuuguuuuggcucuccaucccccucuuguaaagucuuc
......................cguUauauugauucggcucauc.............................................................................................................................................................................................................    1     1     LT-2
......................cgugauauugauucggcucauc.............................................................................................................................................................................................................    31    0     LCK-1
......................cgugauauugCuucggcucauc.............................................................................................................................................................................................................    1     1     LT-1
......................cgugauauugCuucggcucauc.............................................................................................................................................................................................................    1     1     JCK-2
......................cgugauauugauucggcucauU.............................................................................................................................................................................................................    3     1     LT-1
......................Agugauauugauucggcucauc.............................................................................................................................................................................................................    1     1     LT-1
......................cgugauauugauucggcucauc.............................................................................................................................................................................................................    122   0     LT-2
......................cgugauauugauucggcucCuc.............................................................................................................................................................................................................    1     1     LT-2
......................cgugCuauugauucggcucauc.............................................................................................................................................................................................................    2     1     JCK-2
......................cgGgauauugauucggcucauc.............................................................................................................................................................................................................    1     1     LT-2
......................cgugauauugauucggcucauc.............................................................................................................................................................................................................    237   0     LT-1
......................cgugauauugauucggcucauU.............................................................................................................................................................................................................    1     1     JCK-2
......................cgugCuauugauucggcucauc.............................................................................................................................................................................................................    1     1     LT-3
......................cgugauauugauucggcucauU.............................................................................................................................................................................................................    5     1     JCK-3
......................cgugauauugauucggcucauc.............................................................................................................................................................................................................    93    0     JCK-3
......................cgugauauugCuucggcucauc.............................................................................................................................................................................................................    1     1     JCK-1
......................cgugauauugauucggcucauc.............................................................................................................................................................................................................    99    0     JCK-2
......................cguAauauugauucggcucauc.............................................................................................................................................................................................................    1     1     JCK-3
......................cgugauauugauucggcucauc.............................................................................................................................................................................................................    31    0     LCK-2
......................cgugauauugauucggcucauc.............................................................................................................................................................................................................    137   0     LCK-3
......................cgugauauugauucggcCcauc.............................................................................................................................................................................................................    1     1     LT-1
......................cgugauauugauucggcucauc.............................................................................................................................................................................................................    51    0     JCK-1
......................cgugauauugauucggcucauU.............................................................................................................................................................................................................    13    1     LT-2
......................cgugauauugauucggcucauc.............................................................................................................................................................................................................    248   0     LT-3
......................cNugauauugauucggcucauc.............................................................................................................................................................................................................    1     1     JCK-1
......................cgugauauugauucggcucauU.............................................................................................................................................................................................................    1     1     JCK-1
......................cgugauauugauucggcucauU.............................................................................................................................................................................................................    9     1     LCK-3
......................cgugCuauugauucggcucauc.............................................................................................................................................................................................................    1     1     LCK-3
......................cgugauauugauucggcucauUu............................................................................................................................................................................................................    2     1     LT-1
......................cgugauauugauucggcucauUu............................................................................................................................................................................................................    6     1     LT-2
......................cgugauauugauucggcucauAu............................................................................................................................................................................................................    1     1     LT-2
......................cgugauauugauucggcucaucA............................................................................................................................................................................................................    1     1     LCK-3
......................cgugauauugauucggcucaucu............................................................................................................................................................................................................    4     0     JCK-3
......................cgugauauugauucggcucaucu............................................................................................................................................................................................................    34    0     LT-3
......................cgugauauugauucggcucauUu............................................................................................................................................................................................................    1     1     LT-3
......................cgugauauugauucggcucaucA............................................................................................................................................................................................................    2     1     LT-1
......................cgugauauugauucggcucaucA............................................................................................................................................................................................................    3     1     LT-3
......................cgugauauugauucggcucaucu............................................................................................................................................................................................................    17    0     LT-2
......................cgugauauugCuucggcucaucu............................................................................................................................................................................................................    1     1     LT-1
......................cgugauauugauucggcucaucu............................................................................................................................................................................................................    3     0     JCK-2
......................cgugauauugauucggcucauUu............................................................................................................................................................................................................    9     1     LCK-3
......................cgugauauugauucggcucaucu............................................................................................................................................................................................................    1     0     JCK-1
......................cgugauauugauucggcucaucA............................................................................................................................................................................................................    3     1     LT-2
......................cgugauauugauucggcucaucu............................................................................................................................................................................................................    30    0     LT-1
......................cgugauauugauucggcucaucu............................................................................................................................................................................................................    15    0     LCK-3
......................cAugauauugauucggcucaucu............................................................................................................................................................................................................    1     1     LT-2
......................cgugauauugauucggcucaucuU...........................................................................................................................................................................................................    1     1     LT-2
......................cgugauauugauucggcucaucuU...........................................................................................................................................................................................................    2     1     LT-3
......................cgugauauugauucggcucaucuU...........................................................................................................................................................................................................    1     1     JCK-2
......................cgugauauugauucggcucaucuUu..........................................................................................................................................................................................................    1     1     LCK-3
.......................gugauauugauucggcuca...............................................................................................................................................................................................................    1     0     LT-2
.......................gugauauugauucggcucauc.............................................................................................................................................................................................................    2     0     LT-3
.......................gugauauugauucggcucauc.............................................................................................................................................................................................................    1     0     LT-1
......................................................................................cgagccgaaucaauauca.................................................................................................................................................    1     0     LT-2
......................................................................................cgagccgaaucaauaucU.................................................................................................................................................    2     1     LCK-3
......................................................................................cgagccgaaucaauaucac................................................................................................................................................    1     0     LT-1
......................................................................................cgagccgaaucaauaucacu...............................................................................................................................................    5     0     LT-2
......................................................................................cgagccgaaucaauaucacu...............................................................................................................................................    6     0     LT-1
......................................................................................cgagccgaaucaauaucacu...............................................................................................................................................    2     0     LT-3
......................................................................................cgagccgaaucaauaucacuU..............................................................................................................................................    1     1     LT-1
```
